# Fully-automated radiosynthesis of the amyloid tracer [^11^C] PiB via direct [^11^C]CO_2_ fixation-reduction

**DOI:** 10.1186/s41181-019-0065-4

**Published:** 2019-07-16

**Authors:** Pablo Buccino, Eduardo Savio, Williams Porcal

**Affiliations:** 1 Centro Uruguayo de Imagenología Molecular (CUDIM), Av. Dr. Américo Ricaldoni 2010, PC 11600 Montevideo, Uruguay; 2 Departamento de Química Orgánica, Facultad de Química, Universidad de la República, Av. Gral. Flores 2124, PC 11800 Montevideo, Uruguay

**Keywords:** [^11^C] PiB, [^11^C]CO_2_ fixation-reduction, Automated radiosynthesis, PhSiH_3_ / TBAF, Alzheimer’s disease, PET

## Abstract

**Background:**

The β-amyloid radiotracer [^11^C] PiB is extensively used for the Positron Emission Tomography (PET) diagnosis of Alzheimer’s Disease and related dementias. For clinical use, [^11^C] PiB is produced using the ^11^C-methylation method ([^11^C] Methyl iodide or [^11^C] methyl triflate as ^11^C-methylation agents), which represents the most employed ^11^C-labelling strategy for the synthesis of ^11^C-radiopharmaceuticals. Recently, the use of direct [^11^C]CO_2_ fixation for the syntheses of ^11^C-tracers has gained interest in the radiochemical community due to its importance in terms of radiochemical versatility and for permitting the direct employment of the cyclotron-produced precursor [^11^C]CO_2_.

This paper presents an optimised alternative one-pot methodology of [^11^C]CO_2_ fixation-reduction for the rapid synthesis of [^11^C] PiB using an automated commercial platform and its quality control.

**Results:**

[^11^C] PiB was obtained from a (25.9 ± 13.2)% (Average ± Variation Coefficient, *n* = 3) (end of synthesis, decay corrected) radiochemical yield from trapped [^11^C]CO_2_ after 1 min of labelling time using PhSiH_3_ / TBAF as the fixation-reduction system in Diglyme at 150 °C. The radiochemical purity was higher than 95% in all cases, and the molar activity was (61.4 ± 1.6) GBq/μmol. The radiochemical yield and activity (EOS) of formulated [^11^C] PiB from cyclotron-produced [^11^C]CO_2_ was (14.8 ± 12.1)%, decay corrected) and 9.88 GBq (± 6.0%), respectively. These are higher values compared to that of the ^11^C-methylation method with [^11^C]CH_3_OTf (~ 8.3%).

**Conclusions:**

The viability of the system PhSiH_3_ / TBAF to efficiently promote the radiosynthesis of [^11^C] PiB via direct [^11^C]CO_2_ fixation-reduction has been demonstrated. [^11^C] PiB was obtained through a fully automated radiosynthesis with a satisfactory yield, purity and molar activity. According to the results, the one-pot methodology employed could reliably yield sufficiently high tracer amounts for preclinical and clinical use.

**Electronic supplementary material:**

The online version of this article (10.1186/s41181-019-0065-4) contains supplementary material, which is available to authorized users.

## Introduction

The compound 2-(4′-*N*-[^11^C]methylaminophenyl)-6-hydroxybenzothiazole, also known as [^11^C]6-OH-BTA-1 or [^11^C] Pittsburg Compound B ([^11^C]PiB), has long been recognised as a potent PET radiotracer for beta-amyloid (Aβ) plaque imaging in the brains of patients with Alzheimer’s Disease (AD) and other forms of dementia (Engler et al. 2008; Herholz et al. 2007; Klunk et al. 2004; Nordberg 2004, 2008; Rabinovici and Jagust 2009). [^11^C] PiB still remains the gold standard for amyloid imaging in AD diagnosis due to its high affinity for Aβ plaques (K_d_ = 1.4 nM) (Mathis et al. 2003), fast uptake and low non-specific binding.

**Scheme 1:**
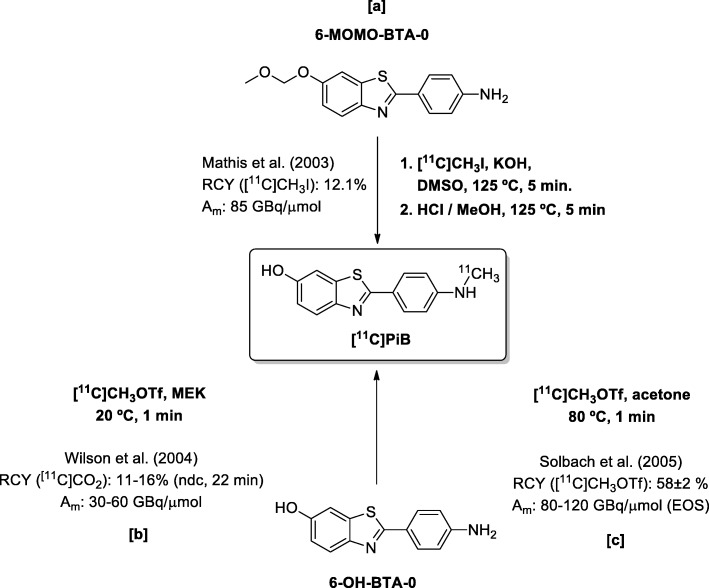
Different radiosyntheses of [^11^C] PiB published in the literature and their parameters

The original radiosynthesis of [^11^C] PiB was performed by Mathis et al. (2003). It consisted of the ^11^C-*N*-methylation of the precursor 2-(4′-aminophenyl)-6-methoxymethoxybenzothiazole (6-MOMO-BTA-0) with [^11^C] methyl iodide ([^11^C]CH_3_I), followed by an acidic deprotection of the methoxymethyl group. The use of the more reactive ^11^C-methylating agent [^11^C] methyl trifluoromethanesulfonate ([^11^C]CH_3_OTf) (Holschbach and Schüller 1993; Jewett 1992) over the unprotected precursor 2-(4′-*N*-aminophenyl)-6-hydroxybenzothiazole (6-OH-BTA-0) has permitted a direct and efficient ^11^C-*N*-methylation, as was demonstrated by Wilson and co-workers (Wilson et al. 2004) and Solbach and co-workers (Solbach et al. 2005) (Scheme 1).

During the last decade, additional optimised radiosyntheses of [^11^C] PiB have been published in the literature. (Philippe et al. 2011; Coliva et al. 2015; Boudjemeline et al. 2017) Although these radiosyntheses proved to be reliable and easily adaptable to a GMP compliant production for patients, all of them use the secondary precursors [^11^C] methyl iodide or triflate. The preparation of these secondary precursors from cyclotron-produced [^11^C]CO_2_ is always time and activity consuming. (Långström et al. 1999). The overall RCY of any [^11^C] PiB radiosynthesis, and subsequently the total activity available for patients, is directly affected by these losses of radioactivity during the preparation of [^11^C]CH_3_I or [^11^C]CH_3_OTf. In this sense, any approach capable of eliminating the steps of synthesis of the ^11^C-methylating agents would be advantageous for a better overall performance in terms of the RCY and the available activity for PET scans.

**Scheme 2:**
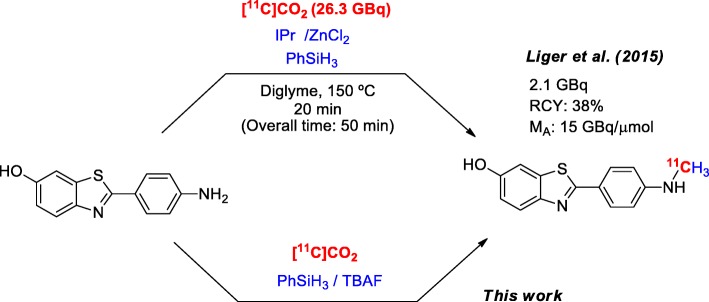
Radiosynthesis of [^11^C] PIB through amine [^11^C] methylation by the direct use of cyclotron-produced [^11^C]CO_2_

[^11^C]CO_2_ is an attractive starting material for radiolabelling because it is produced directly from the cyclotron with a good yield and with a high A_m_. The use of [^11^C]CO_2_ via the so-called ‘fixation’ to synthesise ^11^C-ureas, ^11^C-carbamates, ^11^C-oxazolidinones, ^11^C-carboxilic acids and ^11^C-amides is well-documented in the literature, and it is regarded as ‘a renaissance of PET radiochemistry’ (Rotstein et al., 2013, and references therein). The use of CO_2_ as a C_1_ building block for the methylation of amines has been recognised and demonstrated (Jacquet et al. 2012, 2013; Li et al. 2013; Das et al. 2014). These approaches have been translated to the ^11^C radiochemistry field by Liger and co-wokers (Liger et al. 2015), which constitutes the first experience of using [^11^C]CO_2_ fixation-reduction for the catalytic methylation of amines. In particular, Liger and co-workers used the approach of Jacquet and co-workers (Jacquet et al. 2013), adapting it to an ‘one-pot’ procedure involving PhSiH_3_, 1,3-bis (2,6-diisopropylphenyl)-1,3-dihydro-2*H*-imidazol-2-ylidene (IPr, a *N*-heterocyclic carbene) and ZnCl_2_ in Diglyme at 150 °C. The authors were able to synthesise a series of aromatic and aliphatic amines with an RCY ranging from 24% to 60% (decay corrected, from trapped [^11^C]CO_2_) and also applied the methodology for [^11^C] PiB preparation. [^11^C] PiB was produced after 20 min of incubation at 150 °C (50 min overall time), yielding 2.1 GBq of formulated radiopharmaceuticals (RCY = 38% based on trapped [^11^C]CO_2_ and decay corrected, Scheme 2). Although this work demonstrated the considerable potential of the direct [^11^C] methylation of amines from [^11^C]CO_2_ fixation-reduction, some weaknesses in this methodology when applied to the radiosynthesis of [^11^C] PiB still remains, such as low molar activity (A_m,_ 15 GBq/μmol), long reaction times, carbene stability, metal-catalysed *N*-methylation and few or no description of quality control specifications for the so-prepared batches of radiopharmaceuticals.

With an ageing population, there is an increasing demand for [^11^C] PiB for clinical applications, and therefore the possibility of applying a methodology of [^11^C]CO_2_ fixation-reduction for the routine preparation of [^11^C] PiB to increase its radiochemical yield and thus perform more PET studies for each batch of radiopharmaceuticals was devised. In this context, attention was focused on the strategy recently developed by Liu and co-workers (Liu et al. 2016) for the reductive functionalisation of amines with CO_2_. In their work, the authors explored the utility of the formylation and methylation of amines with CO_2_ using hydrosilanes / TBAF as the catalytic system.

This approach employed mild reaction conditions because it is a metal-free protocol, and the use of the unstable *N*-heterocyclic carbene was avoided.. The radiochemical translation of this methodology was proposed for the radiosynthesis of amines based on radiopharmaceutical interest in particular [^11^C] PiB (Scheme 2).

The aim of this work was to assess the applicability of the PhSiH_3_ / TBAF mediated reductive incorporation of [^11^C]CO_2_ for the radiosynthesis of the β-amyloid tracer [^11^C]PiB. A fully automated, GMP adaptable, fast radiosynthesis of [^11^C] PiB by means of [^11^C]CO_2_ fixation-reduction was developed employing the commercially available platform GE TRACERlab® FX C Pro and its quality control.

## Materials and methods

### Chemicals and materials

All chemicals and reagents used in this work were commercially available products and were used without further purification. Anhydrous solvents MeCN (99.8%), DMF (99.8%), DMSO (99.9%) and bis (2-methoxyethyl) ether (Dyglime, 99.5%) were purchased from Sigma-Aldrich and stored and handled under inert atmosphere. Tetrabutylammonium fluoride (TBAF) 1.0 M in THF and PhSiH_3_ (97%) were acquired from Aldrich, stored in a desiccator and handled in an inert atmosphere (N_2_). 6-OH-BTA-0 was obtained from Siquimia. 4-Toluidine was purchased from Fluka. 6-OH-BTA-0, 6-OH-BTA-1 and 6-(MeO)-BTA-0 were used as analytical standards and were purchased from ABX (GmbH). *N*-methyl-4-toluidine, *N*-formyl-4-toluidine and 2-(4′-*N*-[^11^C]formamidophenyl)-6-hydroxybenzothiazole (PiB *N*-formyl derivative) were synthesised using standard organic chemistry procedures described in the literature (Vogel 1956; Shekhar et al. 2009). Absolute Ethanol (99.8%) was purchased from Merck. Saline and Water for Injection (sterile, USP grade) were acquired from Farmaco Uruguayo. Sep-Pak® C_18_ light cartridges were purchased from Waters and were preconditioned with ethanol (5 mL) followed by water for injection (10 mL) and air (5 mL). Millex® GV sterile filters (0.22 μm, PVDF, 33 mm) were purchased from Millipore. The semipreparative HPLC column used for [^11^C] PiB purification was a Luna® 5 μm C18(2) 100 Å 250 × 10 mm column (Phenomenex). The analytical HPLC column used for both model amine (4-toluidine) ^11^C-methylation and [^11^C] PiB radiosynthesis was a Nucleodur 100–5 C18-ec 250 × 4.6 mm column (Macherey-Nagel). The analytical GC column was a DB-WAX that was 30-m in length, 0.53-mm in diameter and 1.00-mm in film thickness (Agilent).

### Instruments

[^11^C]CO_2_ was produced by the ^14^N(p,α)^11^C nuclear reaction in a PETtrace™ 800 16.5 MeV cyclotron (GE Healthcare). A high-performance target was used for [^11^C]CO_2_ production. The target content was a mixture of N_2_ and 1.0% O_2_ (Praxair). To assess the best labelling conditions, ~ 18.5 GBq of [^11^C]CO_2_ (50 μA, 3 min) were used, and ~ 185 GBq (70 μA, 35 min) were used for complete radiosynthesis (labelling, purification and formulation).

Radiosyntheses were carried out using a TRACERlab® FX C PRO module (GE Healthcare) (see Additional file 1: Figure S1). All valves of the TRACERLab® module were controlled according to the pre-programmed time intervals (time lists) to transfer the reagents from one part to another part of the instruments. Helium pressure was used to transfer the reagents. The transfer of the radioactivity was traced and recorded with an inbuilt radioactivity detector. A by-pass of the iodination loop was made to redirect the purified [^11^C]CO_2_ towards the reaction vessel (reactor). A pre-injection vial (10 mL) was installed before the injection loop, controlled by valve 10 and pressurised with helium from valve 19. The purpose of this vial was to collect the reactor content and the portions of rinse solvent (acetone) added from Vial 3 before the loading of the HPLC loop.

HPLC analyses were performed with a Shimadzu UFLC equipped with UV and a gamma detector (Lablogic Flow RAM HPLC detector). The GC analyses of ethanol, residual reagents and residual solvents were carried out using a Shimadzu GC-2010 Plus equipped with an FID detector. The gamma spectrometry was performed using a 1023-channel Ortec multichannel analyser with a 1″ × 1″ NaI (Tl) crystal. The activity measurements were performed using a Capintec CRC 25 ionisation chamber.

### General procedure for the ^11^C-methylation of amines with [^11^C]CO_2_ using PhSiH_3_/TBAF

#### Preparation of the module

Prior to any radiosynthetic procedure, the molecular sieves (MS, 4 Å, 60–80 mesh) column of the TRACERlab™ FX C Pro was heated to 350 °C under helium flow (40 mL/min) for 15 min and then cooled to room temperature under a helium atmosphere. Simultaneously, the reactor was rinsed with acetone, flushed with helium and further dried under a vacuum to 100 °C for 30 min. The reactor was then cooled to 25 °C and was kept in a positive helium atmosphere (> 300 kPa) until its use in the radiosynthetic experiment.

#### Flushing of the target and lines

To improve A_m_, a protocol described by our group (Savio et al. 2012) was followed. In short, the content of the target was delivered to the molecular sieves column at 350 °C under flowing helium (40 mL/min) to decrease the amount of unlabelled CO_2_ and to send it to waste (‘cold flush’). Immediately before beginning the irradiation for the radiosynthesis, the target was bombarded at 70 μA for 5 min, and its content was directly sent to waste (‘hot flush’).

#### Preparation of the reagent solution

PhSiH_3_ was added slowly over a solution of the amine (4-toluidine or 6-OH-BTA-0) in the desired solvent (0.5 mL) in an inert atmosphere. The solution was vigorously mixed using a vortex agitator, and then TBAF 1.0 M in THF was carefully added over the mixture. The evolution of gas and changes in colour were usually observed during this step. The resulting solution was mixed using a vortex agitator, taken by a syringe, loaded into the reactor of the TRACERlab™ module and sparged with helium (40 mL/min) for 5 min, ideally no more than 5 min before the end of bombardment (EOB).

#### Optimisation of *N*-[^11^C-methyl]-4-toluidine

The cyclotron produced [^11^C]CO_2_ (EOB Activity: A_0_) was sent to the module and trapped in the MS column at room temperature for further purification. The delivery duration was approximately 3 min. The MS column was then heated to 350 °C to desorb purified [^11^C]CO_2_, which was transferred under a helium stream (15 mL/min) to the reactor where the amine solution was placed. Trapping was performed at room temperature. The trapped [^11^C]CO_2_ activity was monitored and registered. Once trapping was complete (a maximum activity A_T_ is reached), the reactor was sealed, and the solution was heated to the chosen temperature. For evaluating the losses of [^11^C]CO_2_ during the heating step, ‘start of labelling activity’ (A_SOL_) was registered once the temperature reached the desired value. Likewise, ‘end of labelling activity’ (A_EOL_) was registered once the labelling time was finished. The solution was cooled to approximately 70 °C and was diluted with 0.5 mL of the same solvent used in the radiolabelling step. The solution was collected in a vial, its activity (A_VIAL_) was measured and radio HPLC analyses were performed to determine the relative radiochemical proportion of the expected species.

#### Automated radiosynthesis of [^11^C]PiB

The same protocol described was conducted using 6-OH-BTA-0 as the precursor amine. Once the labelling step was finished, the solution was cooled and sent to the pre-injection vial. The reactor was then rinsed with acetone (1 mL) from Vial 3, combined with the reaction crude and injected into the HPLC. The separation of [^11^C] PiB was achieved using MeCN:H_2_O (50,50 v/v) at a flow rate of 4 mL/min (t_R_: 8.5–9.5 min). The fraction containing [^11^C] PiB was collected over 50 mL of water for injection and then passed through a Sep-Pak® C18 light cartridge. The excess HPLC solvent was washed with water for injection (10 mL). [^11^C] PiB was eluted from the SPE cartridge with 0.9 mL of absolute ethanol and collected over 5 mL of preloaded saline. In addition, 4 mL of saline were used to rinse the SPE cartridge. The solution of formulated [^11^C] PiB was filtered through a 0.22 μm sterilising filter. The total time of radiosynthesis was about 32 min (since EOB) or 25 min (since the end of [^11^C]CO_2_ trapping).

#### Physicochemical quality control

Radiochemical purity (RCP) was determined using analytical radio-HPLC. An isocratic condition with a CH_3_COONH_4_ / CH_3_COOH buffer 0.1 M, pH = 5.0 and MeCN (40:60 v/v) at a flow rate of 1.2 mL/min was used for 4-toluidine. For [^11^C] PiB, an isocratic condition with H_2_O and MeCN (50:50 v/v) at a flow rate of 1.2 mL / min was used. RCP was calculated considering the area of the peak corresponding to the desired analyte in relation to the sum of the areas of all peaks. The identity of radioactive products was confirmed by co-elution with the non-radioactive standard compounds. UV detection was 270 nm for 4-toluidine and 340 nm for PiB. A_m_ was calculated considering the activity of [^11^C] PiB EOS x RCP in relation to the molar amount of PiB in the sample.

Residual solvents and reagents (such as acetone, acetonitrile, Diglyme and PhSiH_3_) and ethanol were analysed by gas chromatography (GC) in accordance with USP general chapter < 467>. The temperature programme for GC runs was a gradient of 40 °C hold for 2 min, 1 °C/min to 44 °C, 20 °C/min to 200 °C and 200 °C hold for 2 min (total time of 15 min) with helium (11.3 mL/min) as the carrier gas.

The appearance of the solution was checked by visual inspection. The pH level was determined using a calibrated pH-meter. Radionuclidic purity was assessed by recording the corresponding gamma spectrum, and radionuclidic identity was assessed by measuring the physical half-life.

## Results and discussion

### Optimisation of *N*-[^11^C-methyl]-4-toluidine

To assess the potential of the [^11^C]CO_2_ reduction-fixation methodology with PhSiH_3_ / TBAF, the research began by selecting the primary aromatic amine 4-toluidine (4-methylaniline) as a model substrate. The fixation-reduction reaction in different solvents, temperatures and varying amounts of amine substrates and TBAF catalysts was studied using an automated commercial platform TRACERlab™ FX C PRO. The aim was to maintain an excess of PhSiH_3_ (0.6 mmol), which remained constant during the test runs, as well as the total volume of the solution (0.5 mL). Data collected for the radiosynthesis using the reactivity model are summarised in Table 1.

**Table 1:**
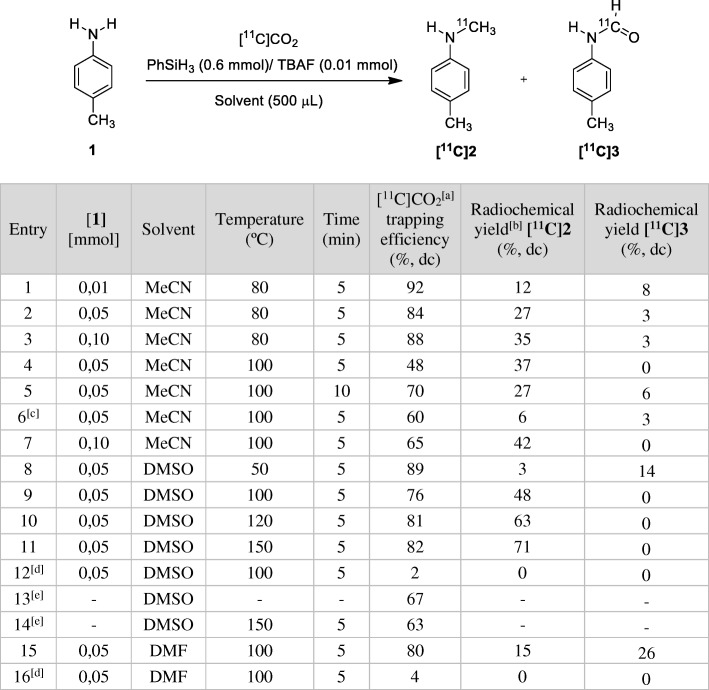
Direct ^11^C-methylation of **1** with [^11^C]CO_2_

[a] Experiments were performed using approximately 18.5 GBq of [^11^C]CO_2_ as a starting activity from the cyclotron; [b] Radiochemical yield (%, dc) was estimated as the actual final activity of **[**^**11**^**C]2 or [**^**11**^**C]3** in relation to trapped [^11^C]CO_2_ (A_T_) (decay corrected); [c] TBAF (0.05 mmol) [d] without TBAF [e] blank experiments in DMSO

The reactivity of the system was evaluated through the radiochemical yield (RCY) of *N*-[^11^C-methyl]-4-toluidine ([^11^C]2). The formation of [^11^C]**2** was observed in practically all conditions where the capture of [^11^C]CO_2_ was considerable. Based on the work of Liu and co-workers (Liu et al. 2016), the experimental conditions began with using MeCN as a solvent. Thus, in MeCN at 80 °C, a radiochemical yield of 14% was obtained for [^11^C]**2** (entry 1, Table 1). An increase in the amount of the amine **1** precursor led to an improvement in the radiochemical yields of [^11^C]**2** (entries 2 and 3). Similar radiolabelling results were obtained when both the temperature and the reaction time were increased (entries 4 and 5). Nonetheless, under these conditions, the RCY observed was lower than 45% in all cases (entries 1–7, Table 1).

Next, DMSO and DMF were selected as solvents for radiolabelling. The choice was inspired by a recent work in which these solvents promoted the *N*-Formylation of amines using carbon dioxide and phenylsilane under mild conditions (Lv et al. 2016). Thus, these solvents might play a key role in promoting the CO_2_ dissolution-insertion and the subsequent interaction of amines with the phenylsilane complexes towards the *N*-methylation reaction. When DMSO was used as a radiolabelling solvent at 150 °C for 5 min, the desired *N-*methylamine [^11^C]**2** was formed with a high radiochemical yield (71%, entry 11, Table 1), presumably due to the higher temperature employed. Indeed, a drop in the RCY for [^11^C]**2** in DMSO was observed as the labelling temperature decreased (entries 8–11, Table 1). It should be noted that the presence of TBAF proved to be essential because the amine [^11^C]**2** was not obtained in its absence (entry 12, Table 1), which is in accordance with the work of Liu and co-workers (Liu et al. 2016). Two blank experiments were carried out in DMSO to determine which species formed in the absence of amine. When [^11^C]CO_2_ was merely collected in a DMSO solution of PhSiH_3_/TBAF (entry 13, Table 1), the formation of a single hydrophilic product eluting at t_R_ = 1.68 min was observed, which was presumed to be [^11^C]HCOO^−^. Upon heating this solution for 5 min at 150 °C (entry 14, Table 1), the predominant radioactive compound eluted at t_R_ = 2.18 min. Based on the proposed reaction mechanism, it was hypothesised that this product could be the key formoxysilane (3) intermediate (See Additional file 1: Scheme S1) because it was the main radiochemical impurity observed in DMSO when amine was present in the labelling crude.

**Fig. 1:**
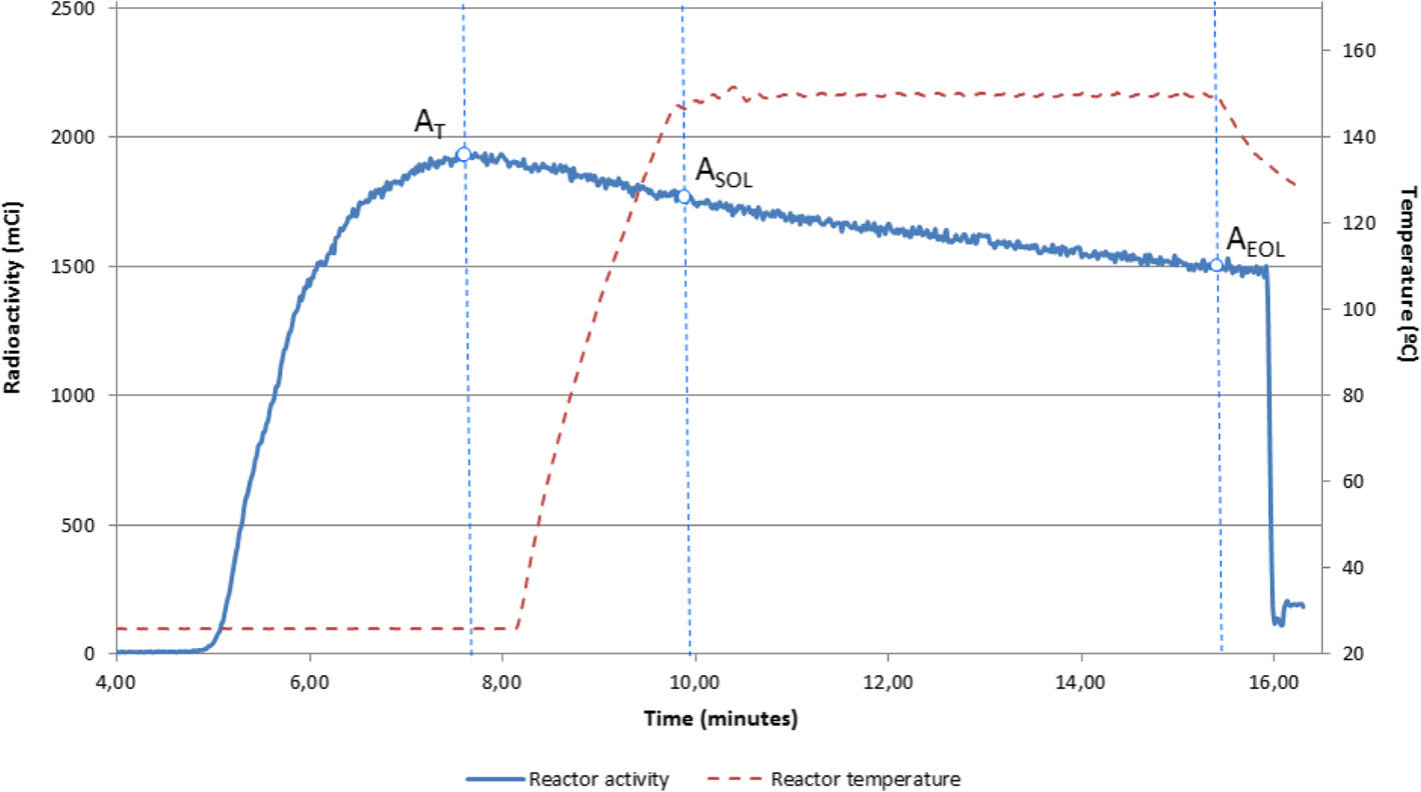
The evolution of the radioactivity and labelling temperature within the reaction vessel. A_T_: maximum [^11^C]CO_2_ radioactivity trapped in the reactor; A_SOL_: Radioactivity at the Start of Labelling; A_EOL_: Radioactivity at the End of Labelling

Finally, a significantly lower radiochemical yield was observed when DMF was tested as a reaction solvent compared to DMSO (entry 15 vs entry 9, Table 1). In some cases, the presence of *N*-[^11^C]-4-tolylformamide ([^11^C]**3**) was detected. This is in agreement with the proposed reaction mechanism in which a *N*-^11^C-formamide would be a precursor for the *N*-^11^C-methyl amine (Additional file 1: Scheme S1). Thus, when DMF was used as a solvent at 100 °C, ([^11^C]**3**) was obtained with an RCY of 26% (entry 15, Table 1), whereas when using MeCN and DMSO as solvents at 80 and 50 °C, [^11^C]**3** was formed in 8 and 14% of RCY, respectively (entries 1 and 8, Table 1).

Another important parameter of the [^11^C]CO_2_ fixation methodology is the trapping efficiency of the reagent solution. This was evaluated as the relationship between maximum trapped [^11^C]CO_2_ activity (A_T_) and [^11^C]CO_2_ EOB theoretical activity (A_0_), and it was decay corrected. The trapping of [^11^C]CO_2_ was dramatically affected by the amount of TBAF in the solution. When no TBAF was present, the trapping efficiency of [^11^C]CO_2_ was less than 5% (entries 12 and 16, Table 1). This is consistent with the mechanism in which TBAF was necessary to form the adduct [PhSiH_3_F]^−^ that would have been responsible for solubilising the [^11^C]CO_2_ in the form of [^11^C]HCOO^−^ (See Additional file 1: Scheme S1); however, increasing the amount of TBAF to 0.05 mmol did not appear to be favourable for a more efficient trapping (60%, entry 6, Table 1). All the solvents used showed good performance for trapping [^11^C]CO_2_ at room temperature when 0.01 mmol of TBAF was used independently from the amine concentration. Nevertheless, when no amine was added (blank runs in DMSO), a small decrease in the trapping efficiency was observed (entries 13 and 14, Table 1).

No losses of radioactivity due to the volatilisation of [^11^C]CO_2_ or other radioactive derivatives were observed during the radiolabelling step, which indicated that the ^11^C species were quantitatively solubilised into the solution (Fig. 1). The losses of radioactivity were evaluated based on the relationship between A_EOL_/A_SOL_ and were decay corrected. In all cases, this relationship was approximately 100%.

### Radiosynthesis of [^11^C]PiB

In accordance with the encouraged results obtained for *N*-[^11^C-methyl]-4-toluidine, DMSO was initially chosen for attempting the labelling of [^11^C] PiB with [^11^C]CO_2_, starting from 0.02 mmol (5.0 mg) of the precursor 6-OH-BTA-0. As shown in Table 2, labelling time varied (entries 1–5, Table 2), and it was found that at 2.5 min, the reductive incorporation of [^11^C]CO_2_ into [^11^C] PiB transcurred with good RCY and radiochemical purity (RCP) (74% and 65%, respectively); even at 1.0 min, both parameters were promising (62% and 61%, respectively). To decrease the amount of 6-OH-BTA-0 to a value closer to those used for the [^11^C]CH_3_OTf labelling methodology, 0.01 mmol (2.5 mg) and 0.005 mmol (1 mg) were used. Lowering the mass of 6-OH-BTA-0 was detrimental for the RCY as well as for RCP when 1 mg was employed (entry 6, Table 2); however, decreasing the molar amount of the precursor proved to be advantageous in terms of A_m_ (entries 2, 7 and 6, Table 2) (as defined in Coenen et al. 2017).

**Table 2:**
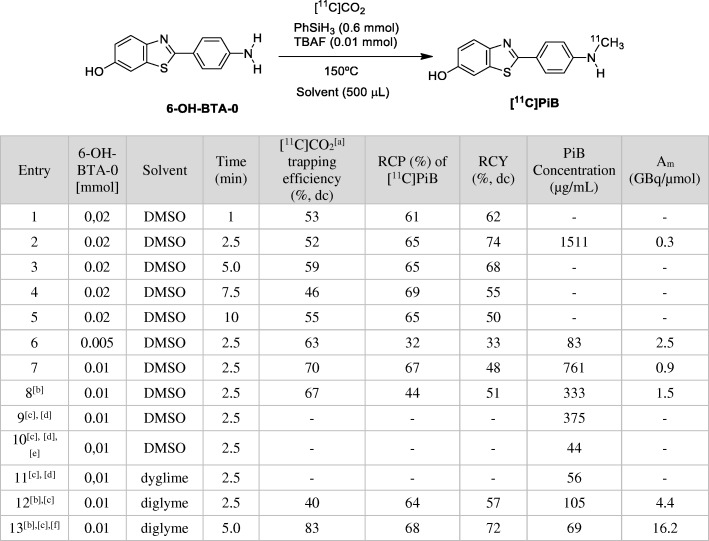
Optimisation of reaction conditions for radiolabelling [^11^C] PIB from direct [^11^C]CO_2_ incorporation

[a] Experiments were performed using approximately 18.5 GBq of [^11^C]CO_2_ as a starting activity from the cyclotron; [b] ‘cold’ and ‘hot’ flushes of targets and lines; [c] solution sparged with He (5 min, 40 mL/min); [d] without [^11^C]CO_2_, [e] without [^11^C]CO_2_ and without PhSiH_3_ / TBAF, [f] *n* = 3

These results could be correlated with a higher degree of reaction between increasing concentrations of the precursor and environmental CO_2_ to obtain PIB (considering an excess of environmental CO_2_ that dilutes [^11^C]CO_2_ during the experiments). In this context, a further flushing of the lines and the target lowered the concentration of unlabelled PiB, but the total concentration was still high for the purposes (entry 8, Table 2). The elevated values in the concentrations of PiB led to the assumption that other phenomena could be added to the incorporation of environmental CO_2_, and in this sense, a contribution of a methyl group from the DMSO used as a solvent was suspected. Indeed, as Jiang and co-workers (Jiang et al. 2014) reported, DMSO can be used as a methylating agent for amines in the presence of HCOOH at 150 °C.

In this system, the presence of unlabelled formoxysilane (PhSiH_2_OCHO) could lead to the activation of DMSO in the form of a methylmethylenesulphonium cation, which could act as the methylating agent depicted by the authors (as shown in Additional file 1: Scheme S2). To demonstrate the contribution of the methyl group from DMSO, two [^11^C]CO_2_ blank experiments were carried out (entries 9 and 10, Table 2). As expected, a higher concentration of PiB was observed in the presence of DMSO and PhSiH_3_/TBAF compared to that obtained when no PhSiH_3_ / TBAF were added to the DMSO solution.

In view of these assumptions, it is proposed that the use of an alternative solvent with a high boiling point might be favourable for improving the A_m_ of [^11^C]PiB. Indeed, the work of Liger and co-workers employed Diglyme as a reaction solvent for the direct [^11^C] methylation of amines from [^11^C]CO_2_ (Liger et al. 2015). When using Diglyme in a [^11^C]CO_2_ blank experiment carried out in the presence of PhSiH_3_ / TBAF, the concentration of PiB decreased considerably with respect to the corresponding experiment with DMSO (entry 11, Table 2).

Similar results were obtained in the presence of [^11^C]CO_2_ in Diglyme at 150 °C for 2.5 min, decreasing the concentration of PiB to 105 μg/mL (entry 12). The RCY and RCP of the [^11^C] PiB achieved under these conditions were 57% and 64%, respectively. Further increasing the labelling time to 5 min allowed for obtaining a higher RCY and RCP for [^11^C] PiB, and the highest A_m_ achieved (entry 13, Table 2). Furthermore, the [^11^C]CO_2_ trapping efficiency was 83% for this condition.

It is important to highlight that the corresponding products of *N*-formylation and *O*-methylation, 2-(4′-*N*-[^11^C]formamidophenyl)-6-hydroxybenzothiazole and 2-(4′-aminophenyl)-6-*O* [^11^C] metoxybenzothiazole, respectively, were not observed under the conditions assayed (data not shown).

### Full radiosynthesis and quality control of [^11^C]PiB

**Fig. 2:**
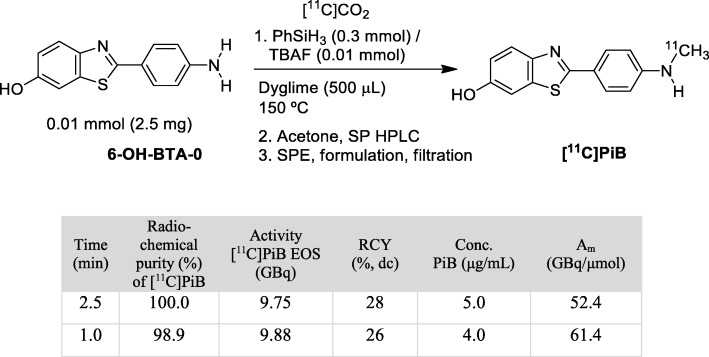
Optimised conditions for the complete radiosynthesis of [^11^C] PiB, starting from 185 GBq of [^11^C]CO_2_ (*N* = 3 each experiment)

**Fig. 3:**
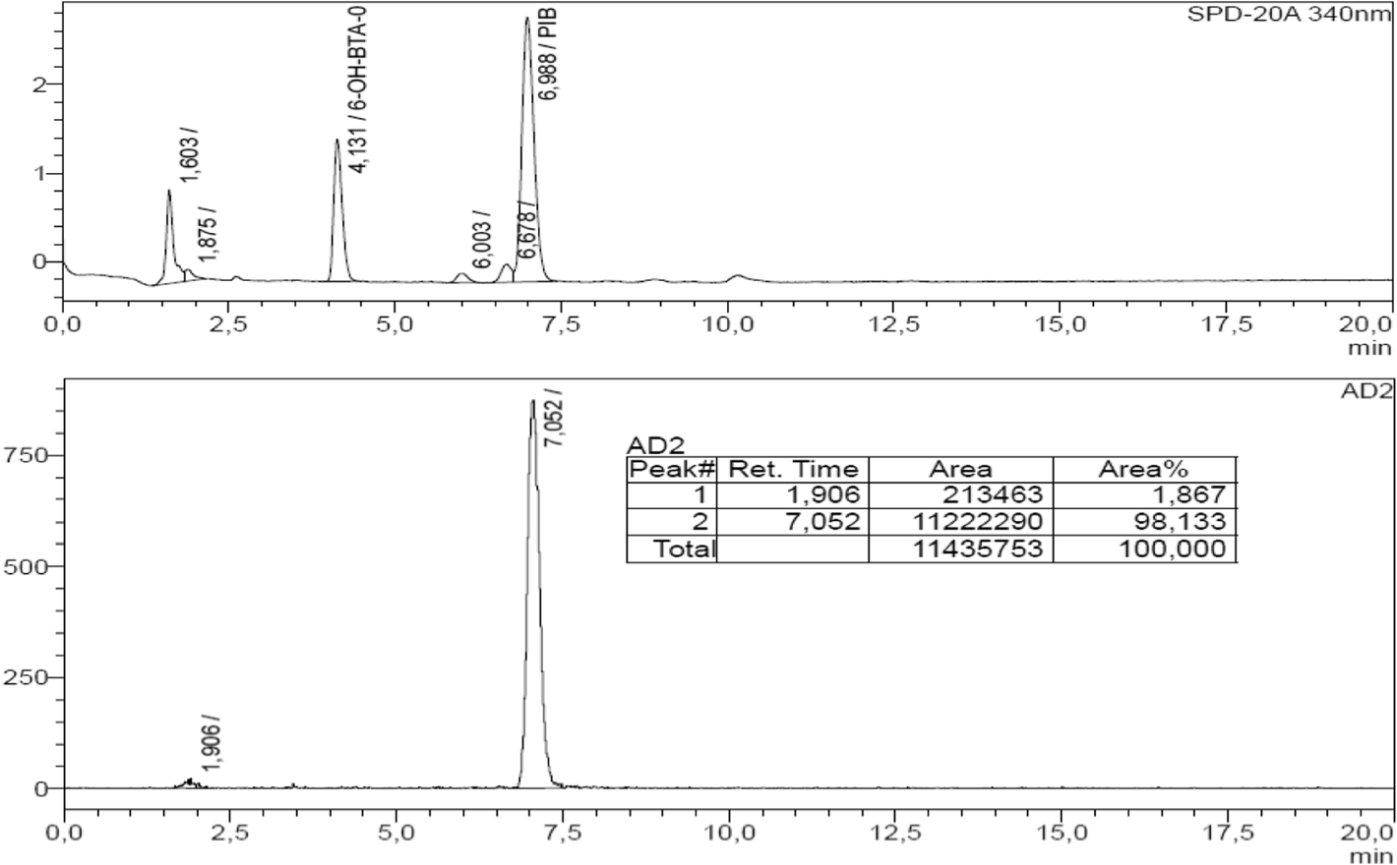
Analytical radio-HPLC for formulated [^11^C]PiB: upper: UV (340 nm); lower: gamma

Based on the results of the previous experiments, the complete radiosynthesis of [^11^C] PiB in Diglyme was tested at 150 °C for 5 min using the starting activities of [^11^C]CO_2_ in the range of 37 to 185 GBq. In general, it was observed that RCY dramatically dropped after the semipreparative HPLC separation, and it was found that this was due to a serious decrease in the radiochemical purity of the [^11^C] PiB formed under these conditions. Indeed, highly lipophilic impurities were found to elute after passing a low polarity mixture through the chromatographic column, such as MeOH:THF (50:50 v/v) (see Additional file 1). Furthermore, an abrupt drop in the trapping efficiency of [^11^C]CO_2_ was noted when 185 GBq of [^11^C]CO_2_ was used as the starting activity. Nevertheless, it was possible to considerably improve the radiochemical yields and molar activities by means of decreasing both the molar amount of PhSiH_3_ and the radiolabelling time. The RCY of 28% and 26%, the A_m_ of 52.4 and 61.4 GBq/μmol and an RCP higher than 95% for [^11^C] PiB were obtained when 0.30 mmol of PhSiH_3_ and labelling times of 2.5 and 1.0 min, respectively, were employed (Figs. 2 and 3). Three consecutive experiments were performed using the optimised radiolabelling condition of 1 min, and physicochemical quality control was performed. An RCP higher than 95% was verified for all the batches (Table 3). In all cases, molar activities were in compliance with the release criteria (> 30 GBq/μmol). Residual solvents were found to be present in quantities below their respective limits, and Diglyme or PhSiH_3_ were not detected in the formulated radiopharmaceutical.

**Table 3:** Physicochemical quality control parameters for three consecutive batches of [^11^C] PiB (labelling time 1 min)

Parameter	Release criteria	1	2	3
Visual inspection	Clear. colourless	Pass	Pass	Pass
pH	4.5–8.5	5.5	5	5.2
Radiochemical identity	Relative retention time: 0.9–1.1	1.02	1.01	1.00
Radiochemical Purity	≥ 95%	100	98.6	98.1
PIB concentration (μg/mL)	No limit stablished	4.61	4.15	4.06
Molar Activity	≥ 30 GBq/μmol	60.3	62.3	61.6
Precursor concentration (μg/mL)	No limit stablished	10.25	3.06	1.63
Total impurities concentration (μg/mL)	No limit stablished	3.18	2.08	1.50
Residual solvents analysis	Acetone: < 0.5%	0.006	0.003	0.056
	Ethanol: < 10%	7.09	7.57	7.67
	Acetonitrile < 0.04%	0.004	0.008	0.005
	Diglyme: < 0.04%	No detected	No detected	No detected
	PhSiH_3_: < 0.04%	No detected	No detected	No detected
Radionucledic identity (t_½_)	19.9–20.9 min	20.4	20.1	20.6
Radionucledic purity	> 99.5% gamma emission at 511 keV	Ok	Ok	Ok

**Fig. 4:**
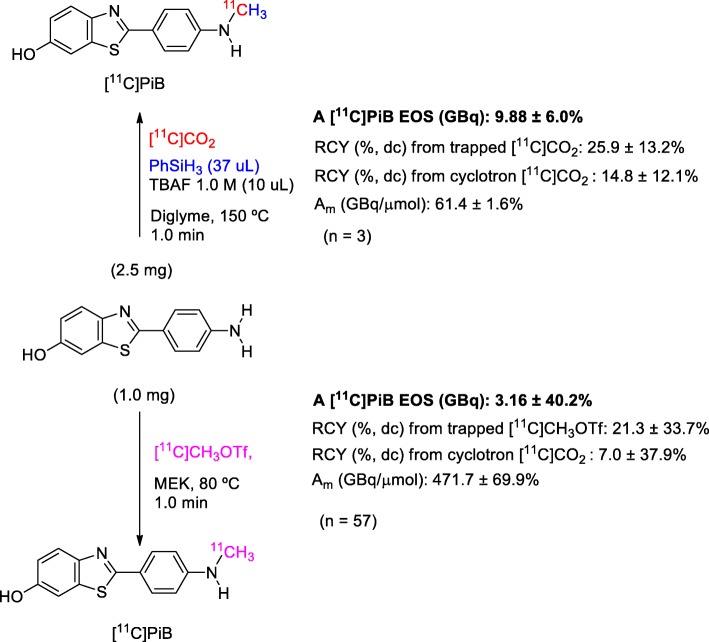
Comparison of the performance of the methodology for the radiosynthesis of [^11^C] PiB using [^11^C]CH_3_OTf and the approach presented in this work employing [^11^C]CO_2_ fixation-reduction with PhSiH_3_ / TBAF. Values are expressed as Average ± Variation Coefficient (%)

To evaluate the applicability of this new approach for the radiosynthesis of [^11^C] PiB, the classical methodology of nucleophilic ^11^C-methylation used in the radiopharmacy laboratory was compared with this novel radiosynthesis from direct [^11^C]CO_2_ incorporation. The ^11^C-methylation with [^11^C]CH_3_OTf, as reported by Phillipe and co-workers (Phillipe et al., Philippe et al. 2011), is currently used, which allows for obtaining an average activity of [^11^C] PiB (EOS) of about 3.14 GBq (RCY 7%, dc) and an A_m_ 471 GBq/umol from ~ 160 GBq of cyclotron-produced [^11^C]CO_2_ (*N* = 57, all productions of the last two years) (Fig. 4). This new approach allows for obtaining a higher activity (more than two-fold) of EOS [^11^C] PiB with a similar overall radiosynthesis time using the same automated module with minimal modifications. As was expected due to isotopic dilution with environmental CO_2_, the direct use of [^11^C]CO_2_ affected the A_m_, though to a degree compatible with the specifications.

## Conclusion

It has been demonstrated that the direct [^11^C]CO_2_ fixation-reduction for the radiosynthesis of [^11^C] PiB can be achieved using the PhSiH_3_ / TBAF system. To obtain knowledge related to the radiochemical nature of the methodology and thus to produce [^11^C] PiB with good and reproducible RCY and A_m_, the influence of physical and radiochemical parameters was investigated. Higher radiochemical yields and activities (EOS) of formulated [^11^C] PiB from cyclotron-produced [^11^C]CO_2_ were obtained compared to that of the ^11^C-methylation method using PhSiH_3_ / TBAF as a fixation-reduction system in Diglyme at 150 °C for 1 min.

Based on these results, a rapid one-pot methodology for the radiosynthesis of [^11^C] PiB by means of the direct use of the primary precursor [^11^C]CO_2_ was developed by employing an automated commercial platform along with a physicochemical quality control proposed for its analysis. In this context, the study indicates the advantages of the unique published work in the application of a direct [^11^C]CO_2_ fixation-reduction methodology for the radiochemical productions of [^11^C] PiB, especially in terms of reaction conditions (carbene- and metal-free), A_m_ and overall radiosynthesis time.

## Additional file

Additional file 1: Semipreparative HPLC chromatogram for [^11^C]PiB. (DOCX 735 kb)

## Data Availability

Please contact author for data request.

## Abbreviations

6-OH-BTA-0: 2-(4′-*N*-aminophenyl)-6-hydroxybenzothiazole
AD: Alzheimer’s Disease
A_m_: Molar activity
dc: decay corrected
Diglyme: Bis (2-methoxyethyl) ether
EOB: End of bombardment
EOS: End of synthesis
GC: Gas chromatography
GMP: Good manufacture practice
HPLC: High-performance liquid chromatography
ndc: no decay corrected
PET: Positron emission tomography
PhSiH_3_: Phenylsilane
PiB: Pittsburg compound B (2-(4′-*N*-methylaminophenyl)-6-hydroxybenzothiazole)
QC: Quality control
RCP: Radiochemical purity
RCY: Radiochemical yield
TBAF: Tetrabutylammonium fluoride
