# Supplementary material for: Fully-automated radiosynthesis of the amyloid tracer [11C] PiB via direct [11C]CO2 fixation-reduction
Source: EJNMMI Radiopharm Chem. 2019 Jul 16;4:14. doi: 10.1186/s41181-019-0065-4 (PMC6635575; doi:10.1186/s41181-019-0065-4)
Supplement: Supplementary file 1 — Semipreparative HPLC chromatogram for [11C]PiB. (DOCX 735 kb) [file 41181_2019_65_MOESM1_ESM.docx]

**FULLY-AUTOMATED RADIOSYNTHESIS OF THE AMYLOID TRACER [^11^C]PIB VIA DIRECT [^11^C]CO_2_ FIXATION-REDUCTION**

Pablo Buccino^1^ (pablo.buccino@cudim.org), Eduardo Savio^1^ (eduardo.savio@cudim.org), Williams Porcal^1,2*^ (wporcal@gmail.com)

**SUPPLEMENTARY INFORMATION**

[1. SUPPLEMENTARY FIGURES AND SCHEMES 2](#_Toc10637486)

[2. Data for Optimization of ^11^C-labeling of [^11^C]PiB 4](#_Toc10637487)

[3. Data for three batches of [^11^C]PiB at 1 min and 2.5 min 4](#_Toc10637488)

[4. Analytical HPLC Standards 5](#_Toc10637489)

[3. Calibration curve for PiB 6](#_Toc10637490)

[4. Analytical HPLC 6](#_Toc10637491)

[5. Semipreparative HPLC chromatogram for [^11^C]PiB 9](#_Toc10637492)

[6. Gas Chromatography 10](#_Toc10637493)

7 Reference…………………………………………………………………………………..13

# SUPPLEMENTARY FIGURES AND SCHEMES


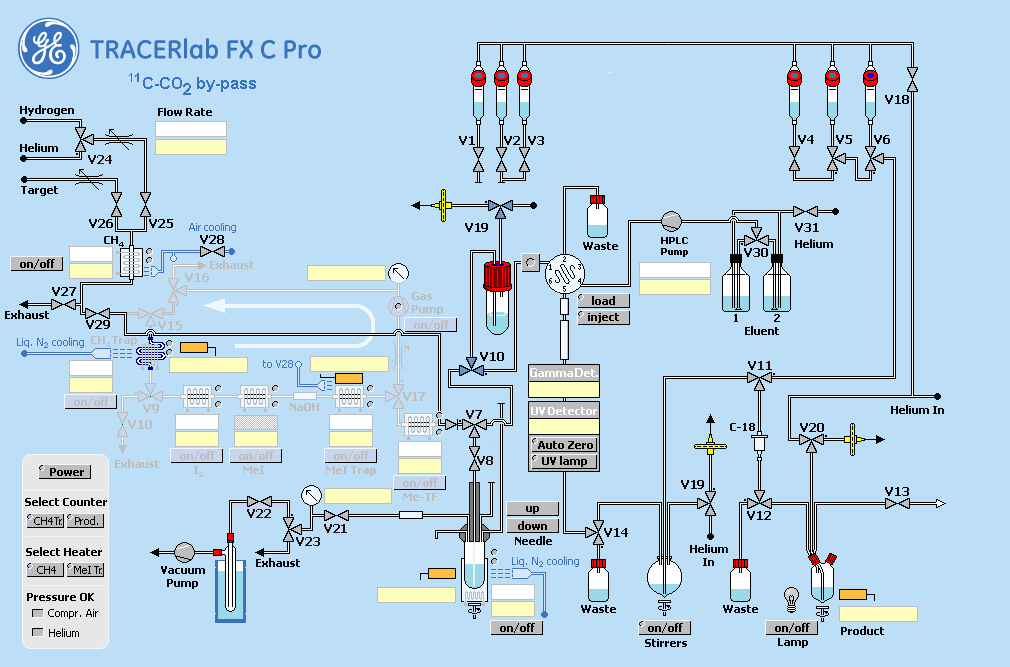


**Figure S1.** Radiosynthetic Platform: GE TRACERlab® FX C Pro with [^11^C]CO_2_ by-pass and pre-injection vial.

**Scheme S1.** [a] Reductive incorporation of CO_2_ into amines promoted by the catalytic system PhSiH_3_ / TBAF , as proposed by Liu and co-workers; [b] Proposed reaction mechanism by Hulla and co-workers.

Mechanistic insights into this process were discussed by the authors and by Hulla and co-workers (Hulla et al., 2016). Two key intermediates in the mechanism are the hypervalent silicon-fluoride intermediate (1) and the formoxysilane (3) (Scheme SUP.1). The first is responsible for the capture of CO_2_ into the solution, whereas the second is responsible for the transference of the formyl group to the amine to form the formamide and silanol as a by-product. Subsequently, the reduction of formamide (4) to methylamine (5) by the silicon-fluoride intermediate (1) is promoted.

**Scheme S2.** Mechanism proposed for the competitive *N*-methylation of 6-OH-BTA-0 from DMSO.

# Data for Optimization of ^11^C-labeling of [^11^C]PiB

| Entry | Amount | Amount TBAF | Time (min) | Starting [^11^C]CO_2_  (mCi) | Radio-chemical purity (%) of [^11^C]PiB | Trapping efficiency | Activity [^11^C]PiB EOS (MBq) | Radio-chemical Yield | Conc. | A_m_ |
| --- | --- | --- | --- | --- | --- | --- | --- | --- | --- | --- |
|  | PhSiH_3_ (mmol) | (mmol) |  | _(EOB)_ |  | (%. dc) |  | (%. dc) | 6-OH-BTA-1 (μg/mL) | (GBq/μmol) |
| 1 | 0.6 | 0.01 | 5 | 5044 | 100 | 51 | 2704.7 | 9 | 2.2 | 33 |
| 2 | 0.3 | 0.01 | 5 | 5044 | 100 | 57 | 5594.4 | 17 | 5.4 | 32.6 |
| 3 | 0.03 | 0.01 | 5 | 5044 | 83.5 | 46 | 2686.2 | 10 | 2.5 | 28.7 |
| 4 | 0.3 | 0.01 | 5 | 2557 | 100 | 69 | 3152.4 | 16 | 5.1 | 18 |
| 5 | 0.3 | 0.01 | 5 | 1135 | 100 | 73 | 1117.4 | 11 | 2.3 | 12 |
| 6^[a]^ | 0.3 | 0.01 | 2.5 | 5044 | 97.1 | 59 | 8587.7 | 25 | 4.7 | 47.6 |
| **7^[b]^** | **0.3** | **0.01** | **2.5** | **5044** | **100** | **57** | **9756.9** | **28** | **5.0** | **52.4** |
| **8^[b]^** | **0.3** | **0.01** | **1** | **5044** | **98.9** | **57** | **9882.7** | **26** | **4.0** | **61.4** |

[a] +0.29 mmol TBAF, +2.5 min 150ºC in order to deprotect [^11^C]6-(OSiH_2_Ph)-BTA-1, [b] *N*=3,

# Data for three batches of [^11^C]PiB at 1 min and 2.5 min, from 185 GBq of [^11^C]CO_2_

| Entry | Time (min) | Radio-chemical purity (%) of [^11^C]PiB | Trapping efficiency  (%. dc) | [^11^C]PiB (GBq) | Radio-chemical Yield  (%. dc) | 6-OH-BTA-1 (μg/mL) | A_m_  (GBq/μmol) |
| --- | --- | --- | --- | --- | --- | --- | --- |
| 1 | 2.5 | 100.0 | 56 | 11.6 | 34 | 6.1 | 51.0 |
| 2 | 2.5 | 100.0 | 58 | 9.25 | 26 | 4.4 | 55.3 |
| 3 | 2.5 | 100.0 | 56 | 8.40 | 25 | 4.4 | 50.8 |
| Average | | **100.0** | **57** | **9.75** | **28** | **5.0** | **52.4** |
| Variation coefficient | | *0.00%* | *1.67%* | *17.10%* | *18.75%* | *19.76%* | *4.85%* |
|  |  |  |  |  |  |  |  |
| 4 | 1.0 | 100.0 | 57 | 10.5 | 30 | 4.6 | 60.3 |
| 5 | 1.0 | 98.6 | 55 | 9.70 | 25 | 4.2 | 62.2 |
| 6 | 1.0 | 98.1 | 59 | 9.40 | 23 | 4.0 | 61.6 |
| Average | | **98.9** | **57** | **9.88** | **26** | **4.0** | **61.4** |
| Variation coefficient | | *1.00%* | *3.47%* | *6.00%* | *13.22%* | *7.35%* | *1.58%* |

# Analytical HPLC Standards


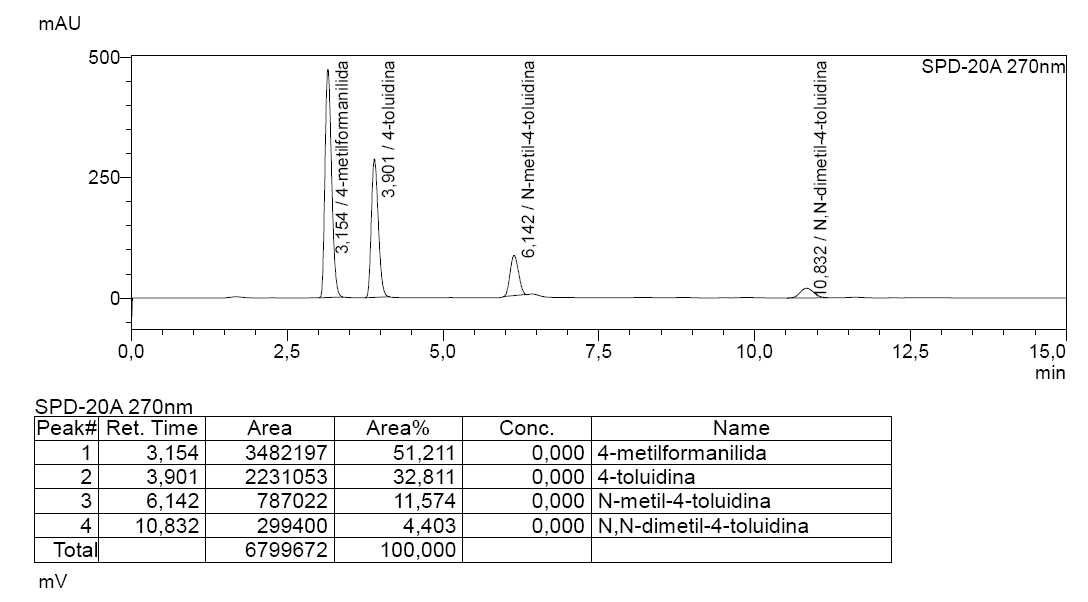


| Compound | t_R_ |
| --- | --- |
| N-(4-tolil)formamide | 3.15 |
| 4-toluidine | 3.90 |
| N-methyl-4-toluidine | 6.14 |
| N,N-dimethyl-4-toluidine | 10.8 |


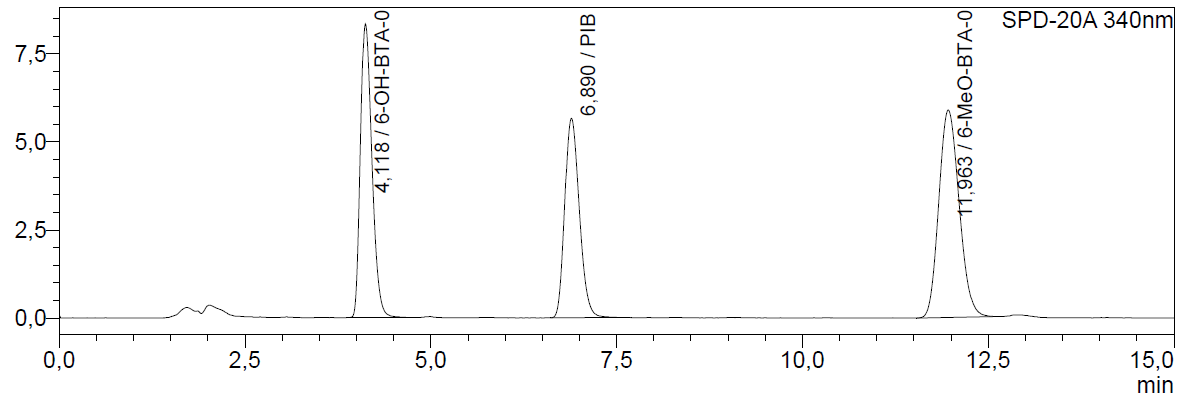


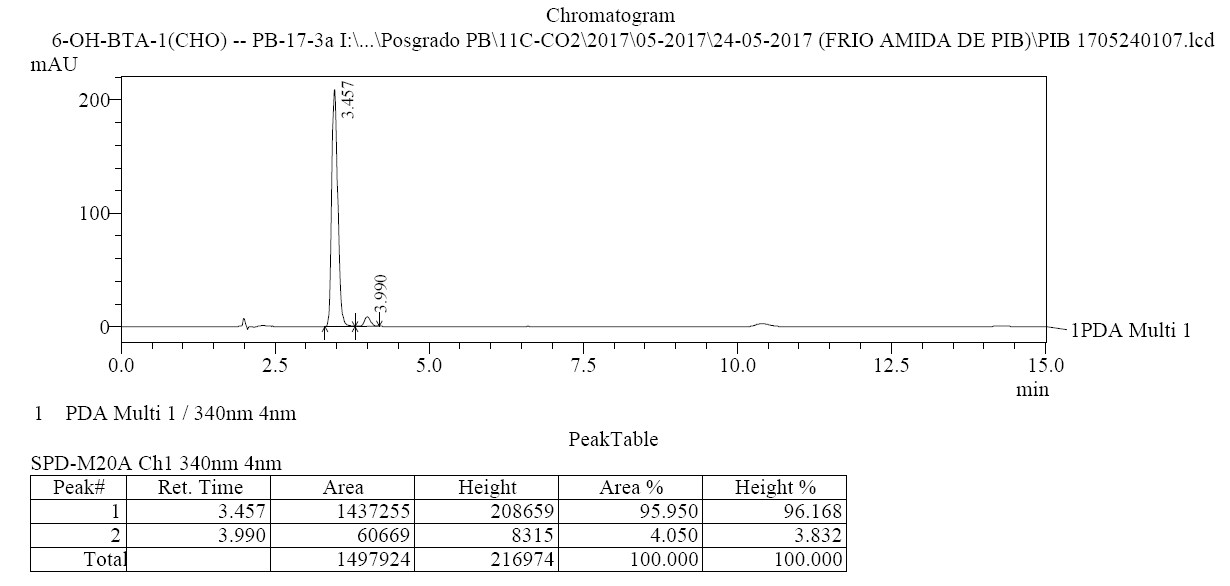


| Compound | t_R_ |
| --- | --- |
| PiB *N*-formamide | 3.45 |
| 6-OH-BTA-0 | 4.12 |
| 6-OH-BTA-1 | 6.89 |
| 6-MeO-BTA-0 | 11.9 |

# Calibration curve for PiB


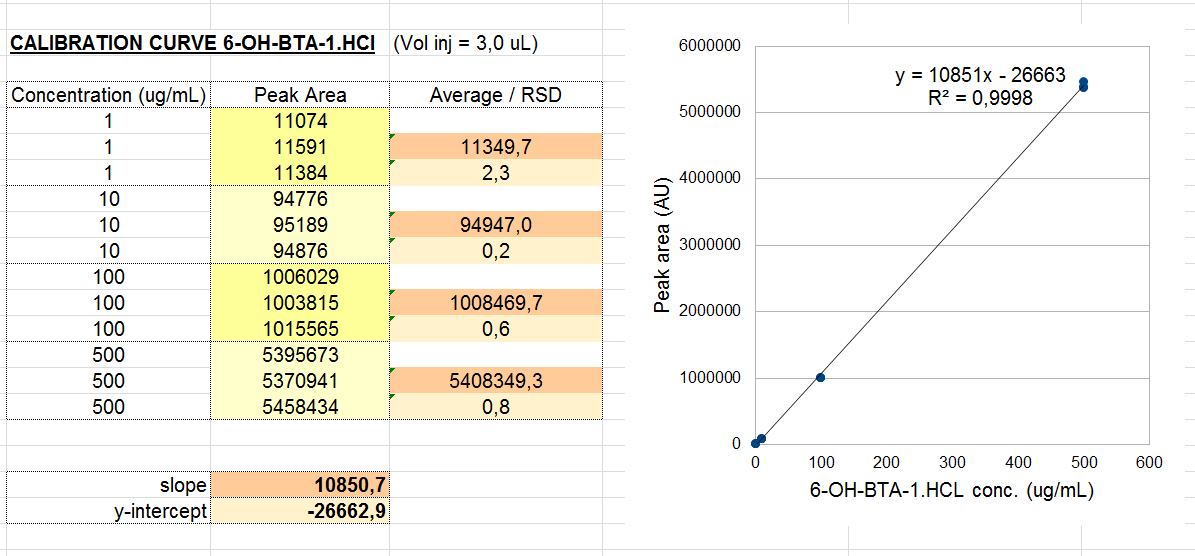


# Analytical HPLC

| - 1. **Analytical gamma HPLC: *N*-[^11^C-methyl]-4-toluidine in MeCN at 100 ºC for 5 min.**   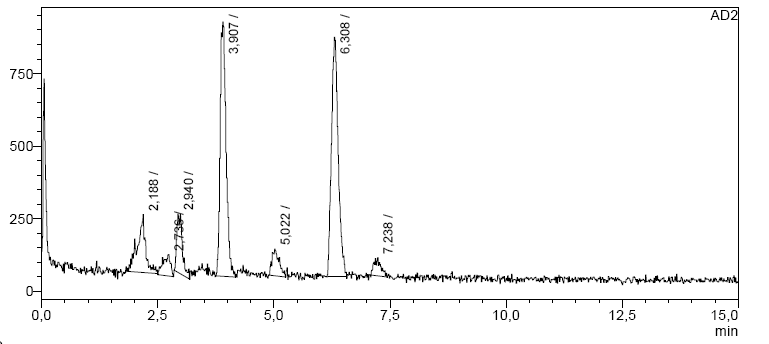  t_R_ for  *N*-[^11^C-methyl]-4-toluidine = 6,3 min, RCP = 37.1%   - 1. **Analytical gamma HPLC: *N*-[^11^C-methyl]-4-toluidine in DMSO at 150 ºC for 5 min.**   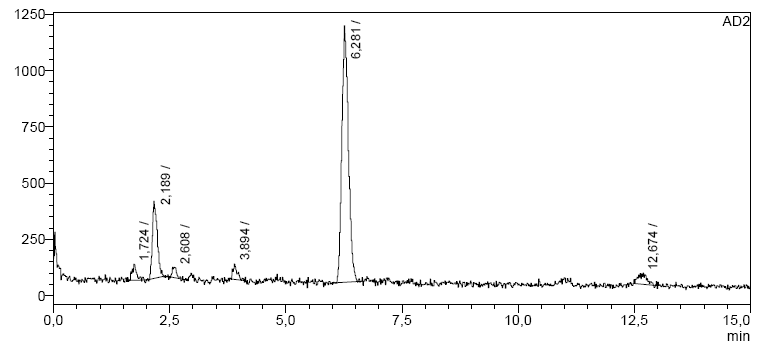  t_R_ for  *N*-[^11^C-methyl]-4-toluidine = 6,3 min, RCP = 72.2%   - 1. **Analytical gamma HPLC: *N*-[^11^C-methyl]-4-toluidine in DMF at 100 ºC for 5 min.**   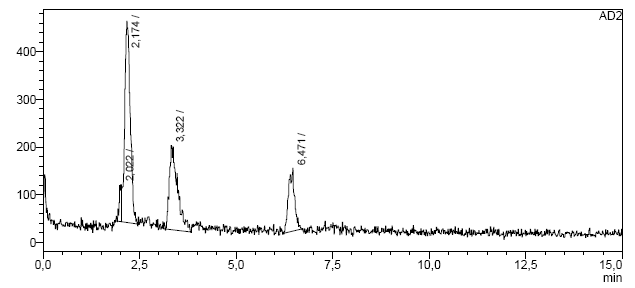  t_R_ for  *N*-[^11^C-methyl]-4-toluidine = 6,4 min, RCP = 30.4% |
| --- |
| - 1. **Analytical gamma and UV HPLC: Optimization of**  **[^11^C]PiB - DMSO 2,5 min at 150 ºC (5 mg) - 180611**   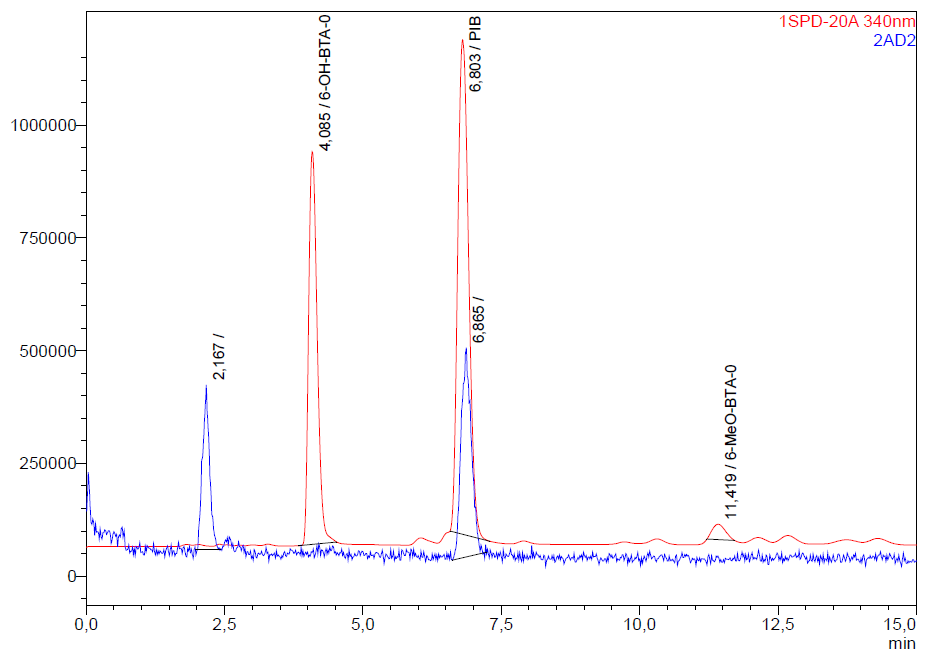  **RCP: 64.6%** |
| - 1. **Analytical gamma and UV HPLC: Optimization of [^11^C]PiB - MeCN 5,0 min at 100 ºC (2.5 mg) - 180831**   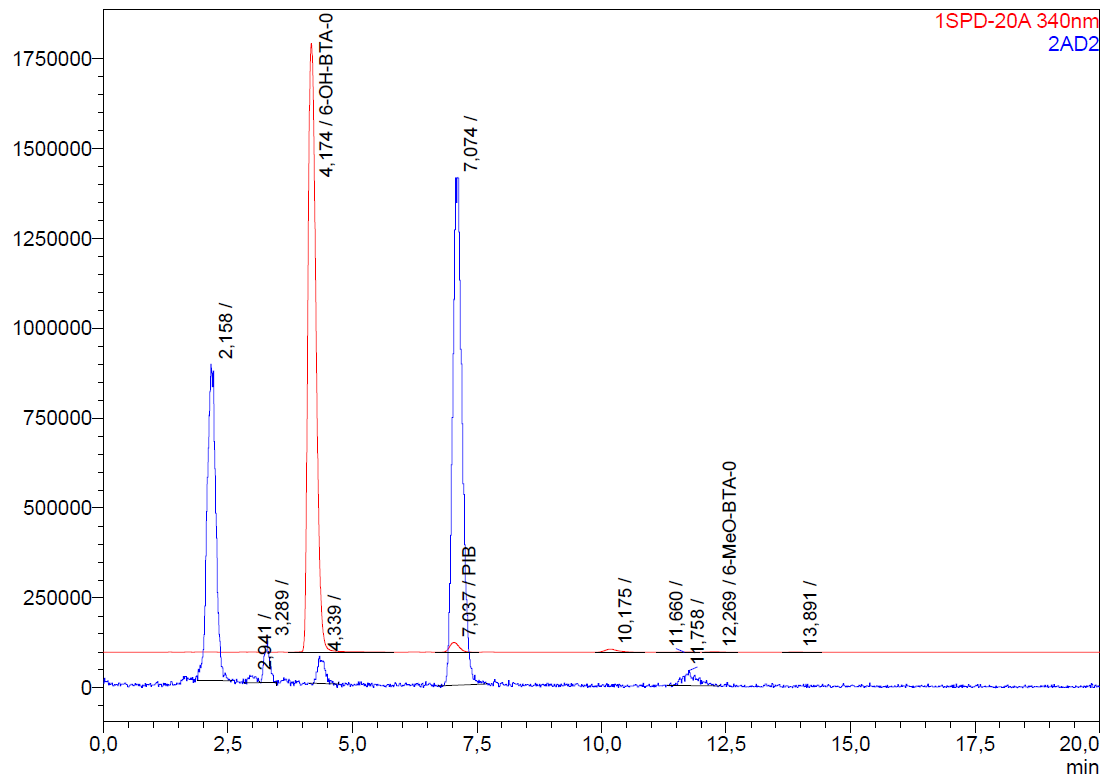  **RCP: 58.9%** |
| - 1. **Analytical gamma and UV HPLC: Optimization of [^11^C]PiB – Diglyme 5,0 min at 150 ºC (2.5 mg) - 180904**   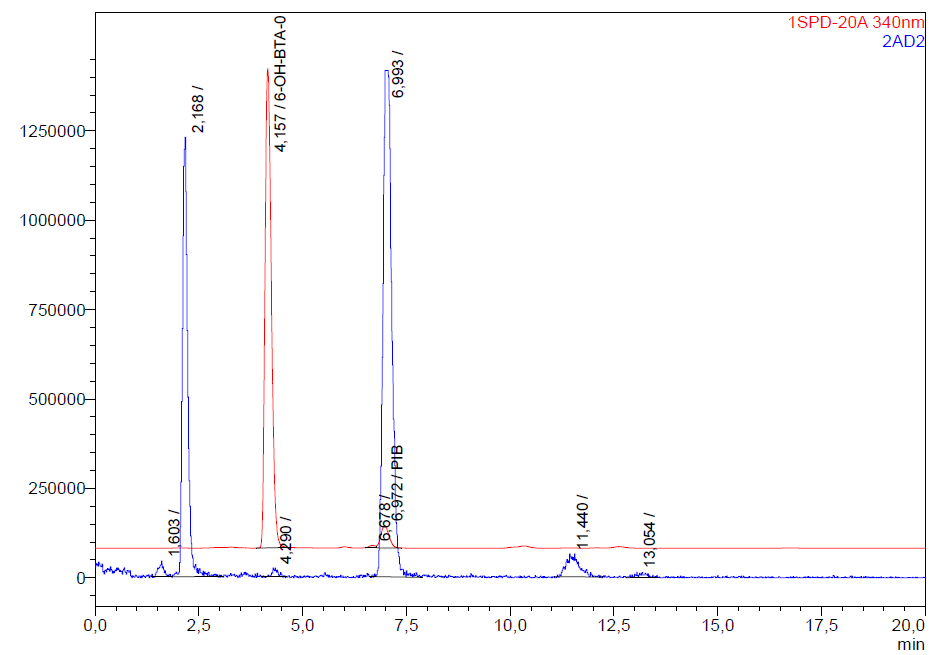  **RCP: 61.7%** |
| - 1. **Complete radiosynthesis of [^11^C]PiB. Diglyme 150 ºC; 2,5 min**   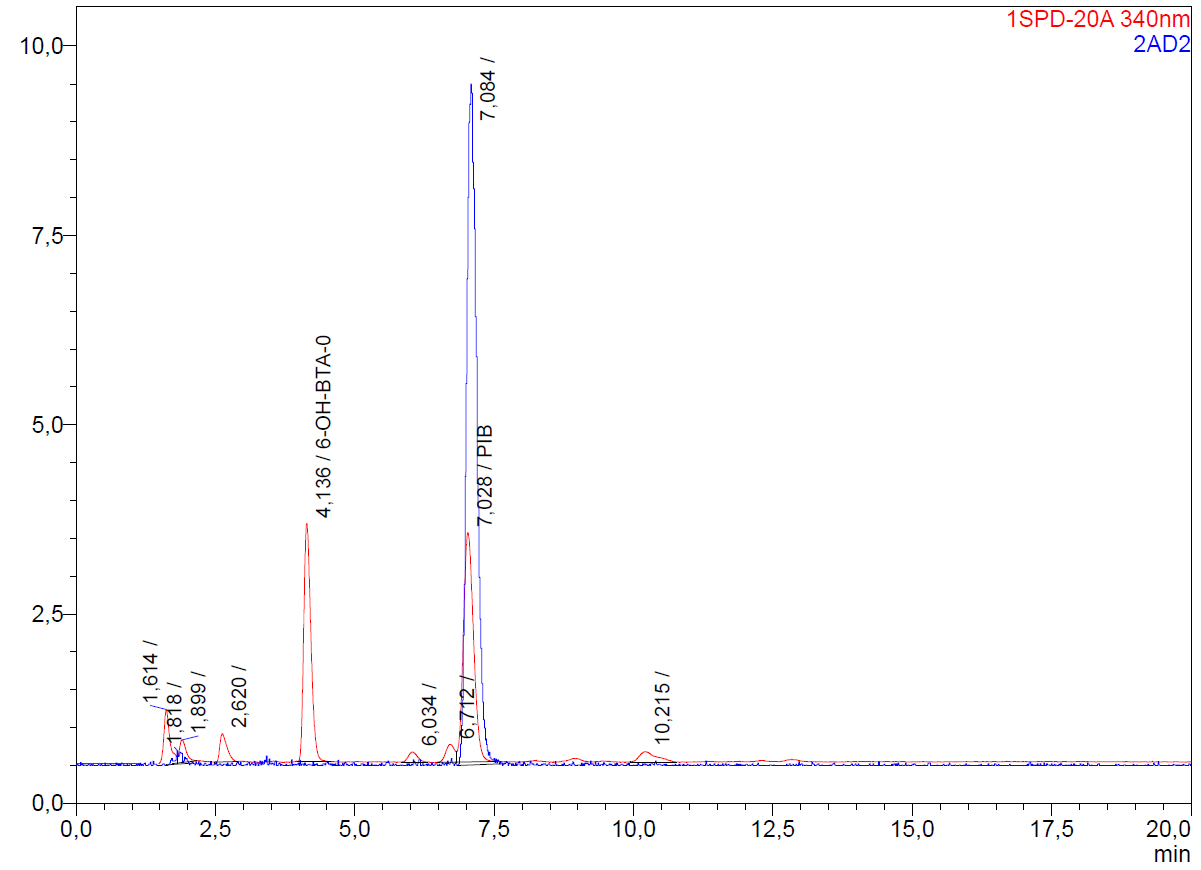   - 1. **Complete radiosynthesis of [^11^C]PiB. Diglyme 150 ºC; 1.0 min**   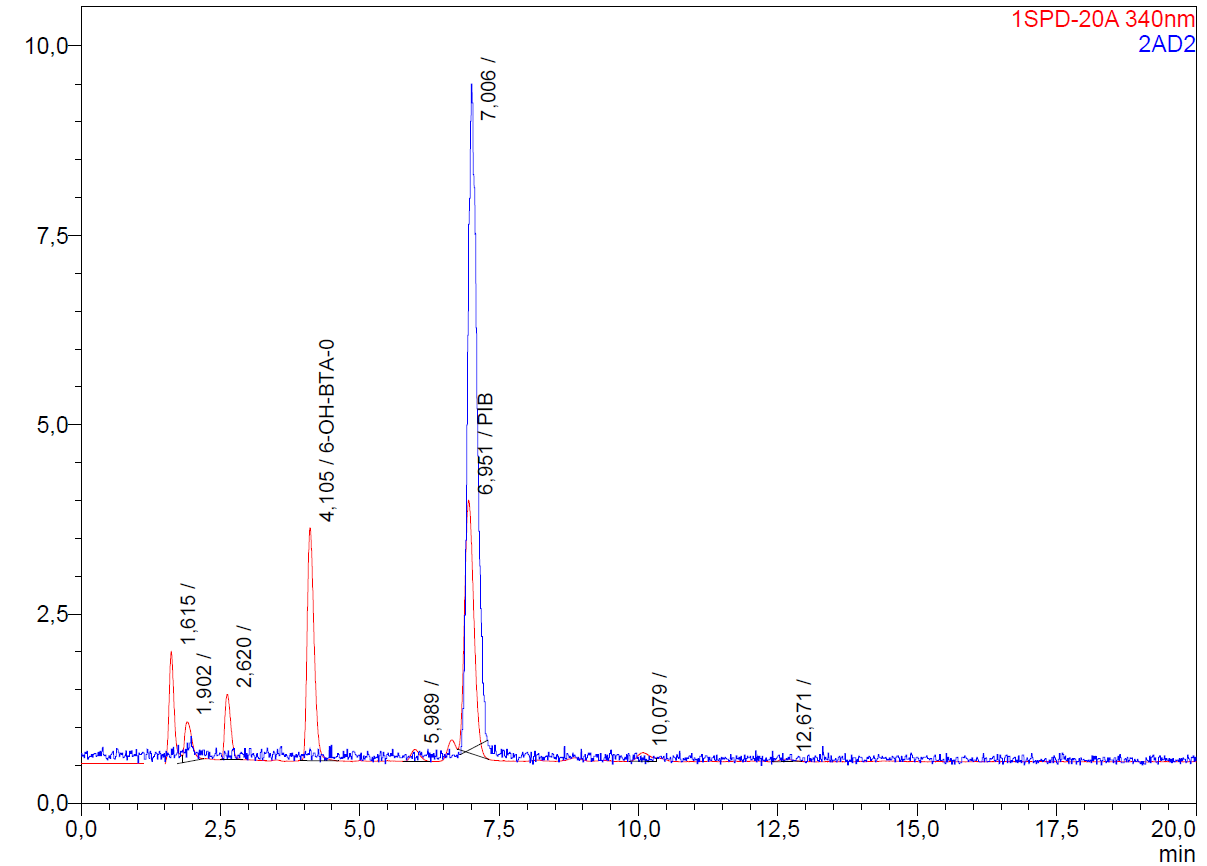 |

# Semipreparative HPLC chromatogram for [^11^C]PiB


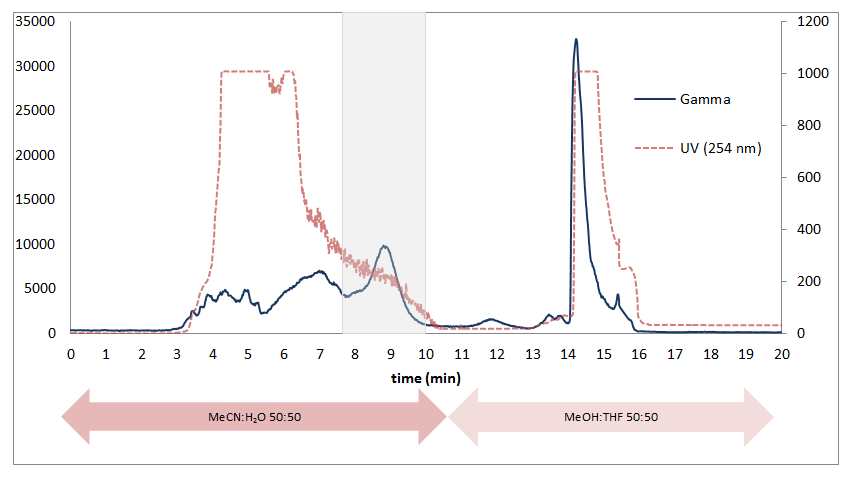


# Gas Chromatography

- 1. Representative GC for one batch of a complete radiosynthesis of [^11^C]PiB (Diglyme, 1 min at 150 ºC)


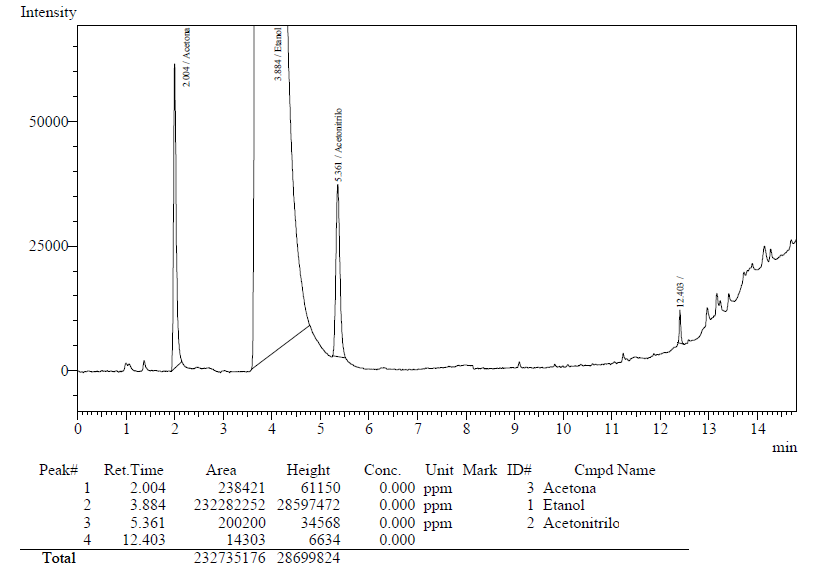


- 1. GC analytical standards


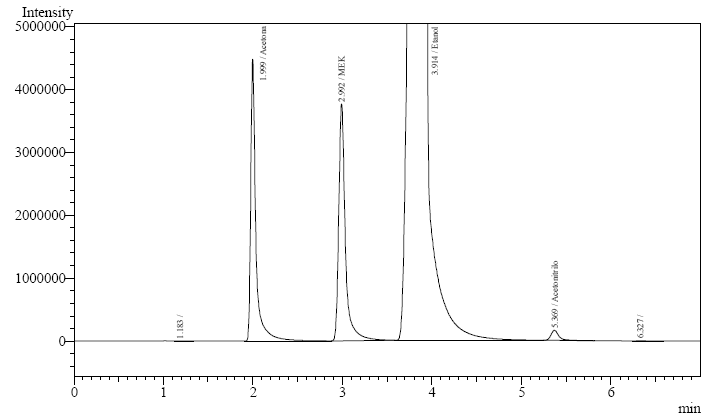


| Compound | t_R_ |
| --- | --- |
| Acetone | 1.99 |
| Methylethylketone | 2.99 |
| Ethanol | 3.91 |
| Acetonitrile | 5.36 |


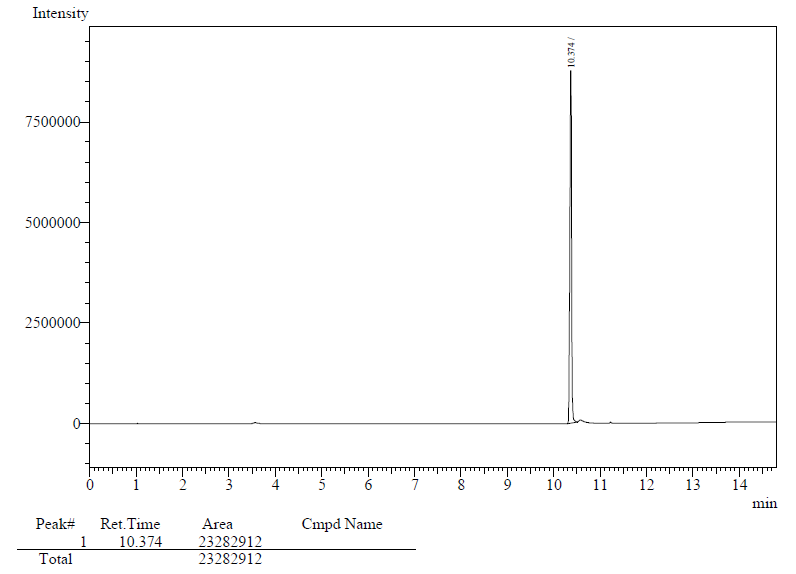


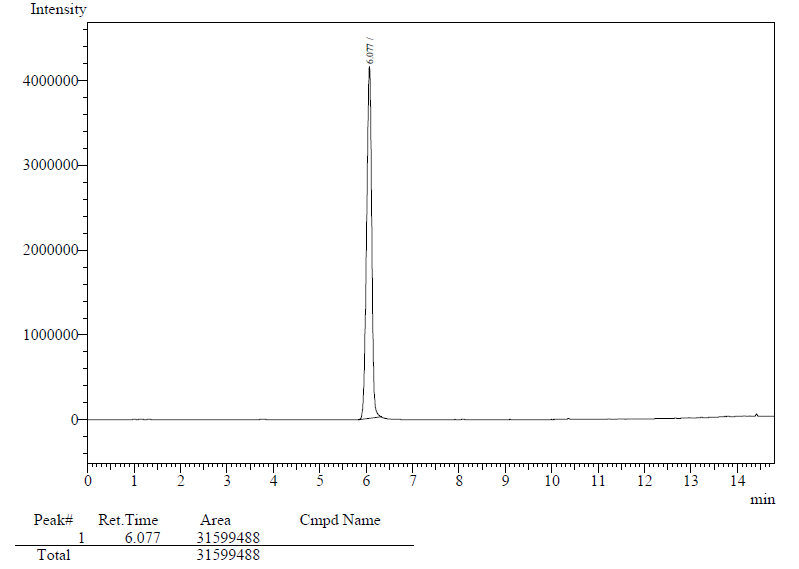


| Compound | t_R_ |
| --- | --- |
| Diglyme | 10.37 |
| PhSiH_3_ | 6.07 |

**Reference.**

Hulla, M., Bobbink, F. D., Das, S., & Dyson, P. J. (2016). Carbon Dioxide Based N‐Formylation of Amines Catalyzed by Fluoride and Hydroxide Anions. *ChemCatChem*, *8*(21), 3338-3342.
